# Core stabilization training improves balance in adolescent male basketball players: a randomized controlled trial

**DOI:** 10.3389/fphys.2026.1887243

**Published:** 2026-07-15

**Authors:** Derya Azim, İbrahim Aras, Ece Diri, Ayşe Nur Tunalı Van Den Berg

**Affiliations:** 1 Faculty of Health Sciences, Department of Physiotherapy and Rehabilitation, Bandirma Onyedi Eylul University, Balikesir, Türkiye; 2 Physiotherapy program, Plato Vocational School, Istanbul Topkapı University, Istanbul, Türkiye; 3 Department of Physiotherapy and Rehabilitation, Institute of Health Sciences, Istanbul Medipol University, Istanbul, Türkiye; 4 Yasam Health LLC- Loop health Care, Chicago, IL, United States; 5 Faculty of Health Sciences, Physiotherapy and Rehabilitation, Istanbul Medipol University, Istanbul, Türkiye

**Keywords:** adolescent male basketball players, balance ability, core stability training, posture, traditional strength training

## Abstract

**Introduction:**

Balance, postural control, and core function are critical for basketball performance and may be affected during adolescence due to rapid growth-related neuromuscular changes. This study examined the effects of a six-week core stabilization training program on postural alignment, balance, and core stability in 12–14-year-old male basketball players.

**Methods:**

Twenty-four male basketball players were randomly assigned to a core stabilization plus traditional strength training group (CST+TST; n = 12) or a traditional strength training group (TST; n = 12). Postural alignment was assessed using the APECS-AI system, static balance with the Balance Error Scoring System, dynamic balance with the Y Balance Test, and core stability with the plank test. Outcomes were measured before and after the intervention. Group × Time effects were analyzed using 2 × 2 mixed-design ANOVA.

**Results:**

Baseline characteristics were similar between groups (p > 0.05). Significant Group × Time interactions were found for static balance (F(1,22) = 26.83, p < 0.001, ηp² = 0.549) and Y Balance Test–Left performance (F(1,22) = 6.10, p = 0.022, ηp² = 0.217), favoring the CST+TST group. No significant Group × Time interactions were observed for Y Balance Test–Right, plank test, or total postural alignment score (p > 0.05).

**Conclusions:**

Six weeks of core stabilization training combined with traditional strength training improved static balance and selected dynamic balance outcomes in adolescent male basketball players. However, it did not provide superior effects on core stability or overall postural alignment. Given that resistance training is already a standard component of preparation for competitive youth basketball players this age, core stabilization exercises may therefore be considered a complementary addition to, rather than a substitute for, an existing traditional strength training program, to enhance balance performance in young basketball players.

## 1 Introduction

Basketball is a high-intensity, multidirectional sport that requires rapid acceleration and deceleration, frequent changes of direction, jumping, landing, and complex footwork. These sport-specific demands depend on well-developed balance, postural control, and trunk stability to maintain movement efficiency and body alignment during offensive and defensive actions (Gong et al., 2024). In adolescent basketball players, these components are particularly important because neuromuscular control, motor coordination, and musculoskeletal development are still progressing (Fransen et al., 2014; Quatman-Yates et al., 2012). The 12–14-year age range is especially relevant because it coincides with peak height velocity in boys, a developmental period characterized by rapid skeletal growth, transient neuromuscular imbalance, reduced coordination, and increased vulnerability to balance deficits and musculoskeletal injury (Borato et al., 2025; Wachholz et al., 2020; Williams et al., 2021).

Core stability plays a central role in postural control and kinetic chain function by facilitating efficient force transfer between the upper and lower extremities (Borghuis et al., 2008; Li, 2023). Within the lumbopelvic–hip complex, local stabilizers and global trunk muscles work synergistically to provide proximal stability for distal mobility (Borghuis et al., 2008). This function is particularly relevant in basketball, where rapid cutting, landing, pivoting, and jumping require precise trunk and lower-extremity control. Targeted core stabilization training may therefore address a developmentally specific vulnerability in adolescent athletes by improving lumbopelvic control, enhancing neuromuscular coordination, and supporting more efficient movement execution (Amato et al., 2025; Schulte et al., 2025).

In the context of the present study, it is important to distinguish between core strength, core endurance, and core stability. Core strength refers to the maximal force-generating capacity of the trunk musculature, whereas core endurance refers to the ability to sustain submaximal trunk muscle contractions over time, which is the capacity assessed by the plank test used in this study (Borghuis et al., 2008). Core stability is a broader dynamic construct that integrates strength, endurance, neuromuscular control, and sensorimotor coordination to resist destabilizing forces and maintain optimal spinal and lumbopelvic alignment during movement (Borghuis et al., 2008; Schulte et al., 2025).

The rationale for expecting balance-related improvements following core stabilization training is based on two complementary mechanisms. First, unstable and perturbation-based core exercises may enhance anticipatory and reactive activation of deep trunk stabilizers, particularly the transversus abdominis and multifidus (Borghuis et al., 2008). Second, improved lumbopelvic control may reduce excessive proximal segment motion during single-leg stance, landing, and directional change tasks, allowing the lower extremity to operate with greater precision (Schulte et al., 2025). This mechanism is especially relevant to dynamic balance performance assessed by the Y Balance Test, where reach distance is strongly constrained by support-limb stability and proximal control (Borghuis et al., 2008; Schulte et al., 2025).

Recent systematic reviews and meta-analyses generally support the beneficial effects of core training on balance, neuromuscular control, and athletic performance (Barrio et al., 2022; Dong et al., 2023; Rodríguez-Perea et al., 2023). Evidence in basketball players also suggests that balance- and core-oriented interventions may improve balance, agility, stability, and skill-related performance, including in adolescent age groups comparable to the present study (Dogan and Savaş, 2021; Gong et al., 2024; Luo et al., 2023; Wang et al., 2025). However, findings remain heterogeneous, including within basketball-specific populations. In young basketball players, core-focused training protocols have not consistently produced statistically superior improvements over comparison interventions in static balance and other physical performance outcomes (Amato et al., 2025), and comparable limited or non-significant effects on isolated trunk endurance measures and static postural alignment have been reported in young athletes following short-duration interventions more broadly (Mueller et al., 2022). This scarcity of basketball-specific evidence in younger adolescent age groups, together with marked variability in training duration, outcome selection, and athlete maturation status across existing studies (Amato et al., 2025; Mueller et al., 2022), indicates that the effects of core training are outcome-specific, population-specific, and dose-dependent, underscoring the need for well-controlled randomized trials specifically targeting younger adolescent basketball populations such as the one examined in the present study.

Resistance training is now a widely recommended and commonly implemented component of preparation for competitive youth athletes, including basketball players in the 12–14-year age range, and is endorsed by professional strength and conditioning organizations as safe and effective when appropriately supervised (Faigenbaum et al., 2009; Gonzalo-Skok et al., 2025). Traditional strength training, used as the active comparator in the present study, may also contribute to trunk function through compound exercises such as squats, deadlifts, and push-ups (Gong et al., 2024). However, traditional strength training primarily emphasizes global force production, whereas targeted core stabilization training is designed to improve local stabilizer function, lumbopelvic control, and neuromuscular coordination (Pan and Wei, 2022). Therefore, comparing core stabilization combined with traditional strength training against traditional strength training alone allows the additive contribution of core-specific training to be examined beyond the general strength stimulus received by both groups.

Despite growing evidence supporting core training in athletic populations, three important gaps remain. First, most existing studies have examined adult or mixed-age athletes, while evidence specific to 12–14-year-old basketball players remains limited (Gong et al., 2024). This age range is particularly important because it coincides with peak height velocity, a developmental window characterized by rapid skeletal growth, transient neuromuscular imbalance, and heightened vulnerability to balance and movement-control deficits (Borato et al., 2025; Wachholz et al., 2020; Williams et al., 2021); core training effects established in older or skeletally mature athletes may therefore not generalize to this distinct and especially injury-prone developmental stage. Second, although balance outcomes are frequently reported, few studies have included postural alignment as an outcome, leaving unclear whether short-term core stabilization training can produce measurable changes in spinal and segmental alignment (Pan and Wei, 2022). Third, despite the widespread use of traditional strength training in youth basketball preparation (Faigenbaum et al., 2009; Gonzalo-Skok et al., 2025), few randomized trials have directly tested whether adding core stabilization training to an existing strength training program provides additive benefit beyond traditional strength training alone, particularly using a comprehensive, multidomain outcome battery capable of capturing postural alignment, static balance, dynamic balance, and core endurance within the same cohort.

Therefore, this randomized controlled trial aimed to investigate the effects of a six-week core stabilization exercise program, implemented in addition to traditional strength training, on postural alignment, static balance, dynamic balance, and core endurance in 12–14-year-old male basketball players. To the best of our knowledge, this is the first randomized controlled trial to compare core stabilization plus traditional strength training with traditional strength training alone in this specific developmental age group using a comprehensive multidomain assessment battery that included postural alignment evaluation through the APECS system. The primary hypothesis was that adding a structured core stabilization program to traditional strength training would result in significantly greater improvements in static balance, dynamic balance, and core endurance compared with traditional strength training alone.

## 2 Materials and methods

### 2.1 Study design

This study was designed as a randomized controlled pre–post intervention study to examine the effects of a core stabilization exercise program on postural alignment, balance, and core stability in adolescent male basketball players. The study included two groups: a core stabilization plus traditional strength training group (CST+TST) and a traditional strength training group (TST). Throughout this manuscript, the intervention group is consistently referred to as the CST+TST group, as participants in this group received both the core stabilization program and the traditional strength training program. All participants were assessed before and after the intervention period using standardized posture, balance, and core stability assessments.

### 2.2 Participants

**Figure 1:**
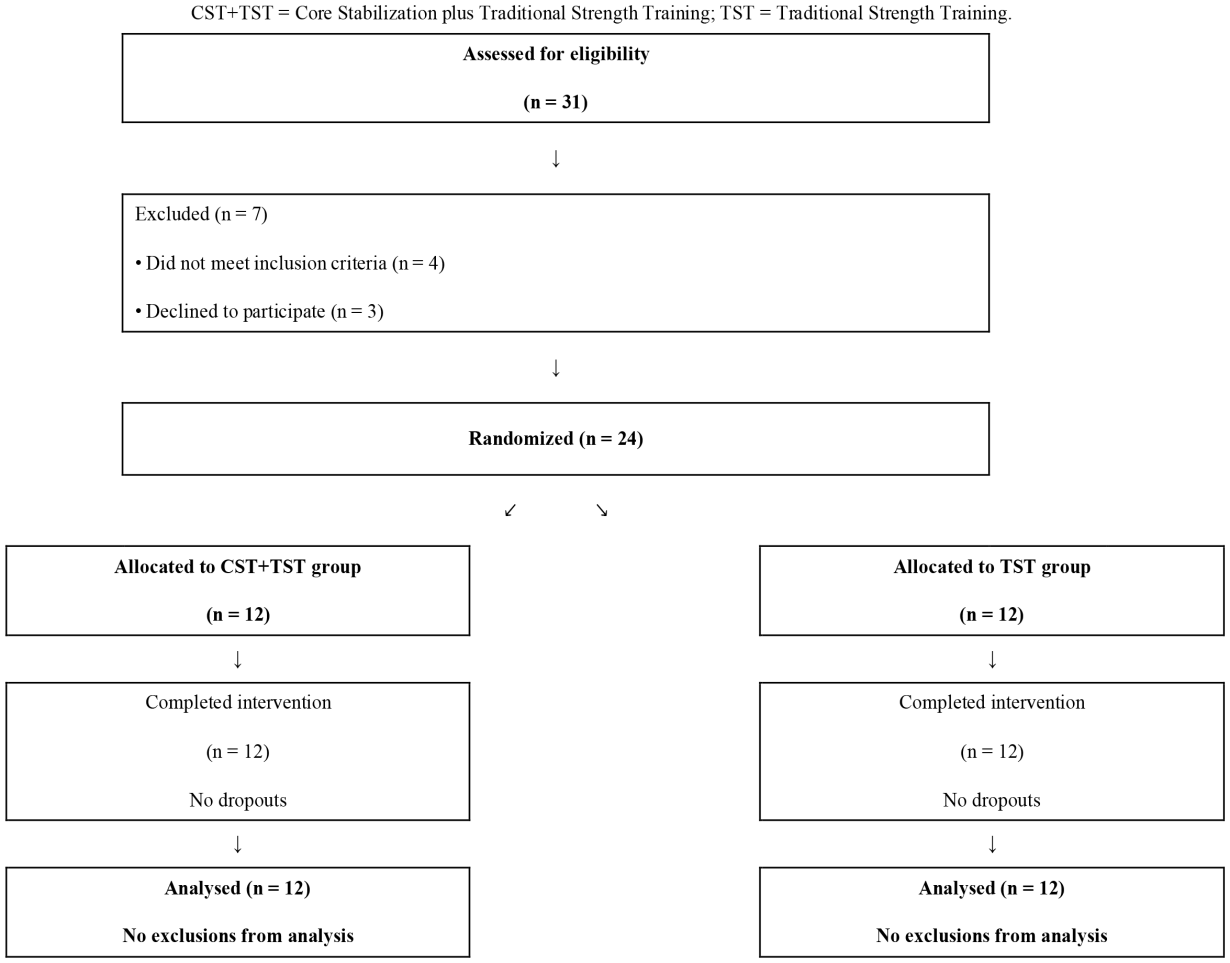
Consort flow diagram. CST+TST, Core stabilization plus traditional strength training; TST, Traditional strength training.

A total of 24 male basketball players aged 12–14 years were included in the study. Participants were recruited on a voluntary basis from Bordo Sports Club and surrounding basketball clubs. All participants were competitive, club-affiliated youth basketball players who trained regularly with their club squads and competed in organized regional youth league fixtures; participants were not recreational or casual players, but none held representative status at national-team or elite academy level. All participants were registered with their respective clubs and had a mean playing experience of approximately 2–3 years. During eligibility screening, 31 athletes were assessed; four did not meet the inclusion criteria and three declined to participate. Consequently, 24 athletes were enrolled and randomized. Participants were allocated into two groups: the core stabilization training group (n = 12) and the traditional strength training group (n = 12). The flow of participants throughout the study is presented in **Figure 1** using a CONSORT flow diagram. The individual age of every enrolled participant in both groups fell within the specified 12–14-year range (CST+TST: 12.75 ± 0.87 years; TST: 12.92 ± 0.79 years; **Table 1**), confirming full compliance with the age-based eligibility criterion.

**Table 1:** Baseline characteristics of the groups.

Variable	CST + TST group	TST group	t-value	p-value	Cohen’s d
Age, years	12.75 ± 0.87	12.92 ± 0.79	-0.49	0.628	-0.20
Height, cm	167.17 ± 12.74	166.83 ± 7.77	0.08	0.939	0.03
Body mass, kg	59.42 ± 16.53	59.67 ± 10.77	-0.04	0.965	-0.02

Values are presented as mean ± standard deviation. Independent samples t-test was used to compare baseline characteristics between groups. CST+TST, Core Stabilization plus Traditional Strength Training; TST, Traditional Strength Training only. Cohen’s d was calculated using the pooled standard deviation. Individual ages ranged from 12.1 to 14.0 years in the CST+TST group and from 12.0 to 13.9 years in the TST group, consistent with the study’s 12–14-year inclusion criteria.

The inclusion criteria were as follows: being a male basketball player aged 12–14 years, having no history of serious injury or surgery within the previous six months, volunteering to participate in the study, and providing written parental informed consent. Athletes were excluded if they had orthopedic, neurological, or cardiovascular disorders, balance-related conditions such as vertigo, or previous experience with a structured core stabilization training program. Biological maturation status was not assessed using objective methods such as Tanner staging or peak height velocity estimation in the present study; this represents one of the methodological limitations of the study.

This study was designed as an exploratory pilot randomized controlled trial conducted without an *a priori* power calculation before data collection. All 24 enrolled participants completed the six-week intervention period, and no dropouts occurred. Accordingly, all participants were included in the final analyses (CST+TST: n = 12; TST: n = 12). To characterize the sensitivity of the study design, a *post-hoc* power analysis was performed using G*Power software. For the primary outcome, the Balance Error Scoring System, the observed power for the significant Group × Time interaction was 0.90 (F(1,22) = 26.83; α = 0.05).

### 2.3 Randomization and allocation concealment

Following baseline measurements, participants were randomly assigned in a 1:1 ratio to either the core stabilization plus traditional strength training group (CST+TST) or the traditional strength training group (TST). A computer-generated randomization sequence was used to determine group allocation. Participants were first assigned unique identification codes, and these codes were randomly allocated to one of the two intervention conditions using a random number generator (Microsoft Excel RAND function). Allocation concealment was ensured by placing the randomization assignments in sequentially numbered, sealed, opaque envelopes, which were opened only after completion of the baseline assessments. This procedure ensured that the research team had no prior knowledge of the upcoming group allocation and thereby minimized the risk of selection bias. Because randomization was conducted after baseline testing, allocation could not influence baseline assessment procedures.

The CST+TST group received a structured core stabilization exercise program combined with traditional strength training, whereas the TST group performed the traditional strength training program alone, with no additional core stabilization component. All outcome measurements were performed using the same standardized procedures at pre-test and post-test. Regarding blinding, participants could not be blinded to their group allocation given the nature of the intervention. However, the assessors who conducted the outcome measurements (BESS, Y Balance Test, Plank Test, and APECS postural assessment) were kept unaware of each participant’s group assignment throughout the data collection process. This single-blind design was implemented to reduce the risk of observer bias in observational assessments, particularly for the BESS and Plank tests, which require direct scoring of performance quality and balance errors.

### 2.4 Intervention program

The study included two intervention arms: a core stabilization plus traditional strength training group (CST+TST) and a traditional strength training group (TST). Participants in the CST+TST group performed core stabilization training combined with traditional strength training, whereas participants in the TST group performed traditional strength training alone.

**Figure 2:**
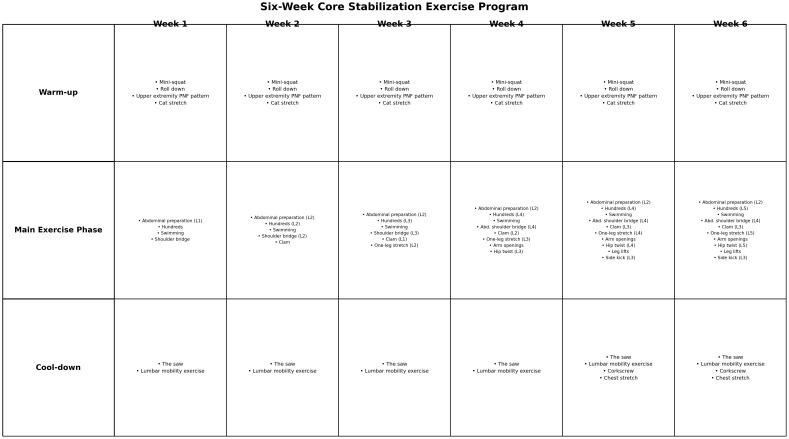
Six-week core stabilization exercise program. Each session was administered three days per week and lasted approximately 45–50 minutes, consisting of a 10-minute warm-up, a 30-minute main exercise phase, and a 5-minute cool-down. Exercise difficulty and the number of repetitions were progressively increased each week according to athletes’ adaptation and tolerance. Exercises are listed in their order of performance within each session. PNF, proprioceptive neuromuscular facilitation.

Participants in the CST+TST group performed a structured core stabilization exercise program in addition to traditional strength training. The core stabilization program was delivered three days per week and targeted the trunk and lumbopelvic region through progressively advanced stabilization exercises. Each session lasted approximately 45–50 minutes and consisted of a 10-minute warm-up, a 30-minute main exercise phase, and a 5-minute cool-down. The warm-up phase included mini-squats, roll-down exercises, upper-extremity proprioceptive neuromuscular facilitation patterns, and cat stretch exercises. The main phase included abdominal preparation, hundreds, swimming, shoulder bridge, clam, one-leg stretch, arm openings, hip twist, leg lifts, and side kick exercises. The cool-down phase consisted of the saw, lumbar mobility exercises, corkscrew, and chest stretching exercises (**Figure 2**).

Exercise progression was applied by increasing task complexity rather than session duration. During Weeks 1–2, exercises were performed in their basic forms, with emphasis on correct technique, postural control, and breathing. During Weeks 3–4, lever length was increased and single-limb loading was introduced where appropriate. During Weeks 5–6, selected exercises were performed on unstable surfaces, such as foam pads, and combined with multidirectional limb movements. Progression was individualized according to each athlete’s technical execution, adaptation, and tolerance, as determined by the supervising physiotherapist.

**Figure 3:**
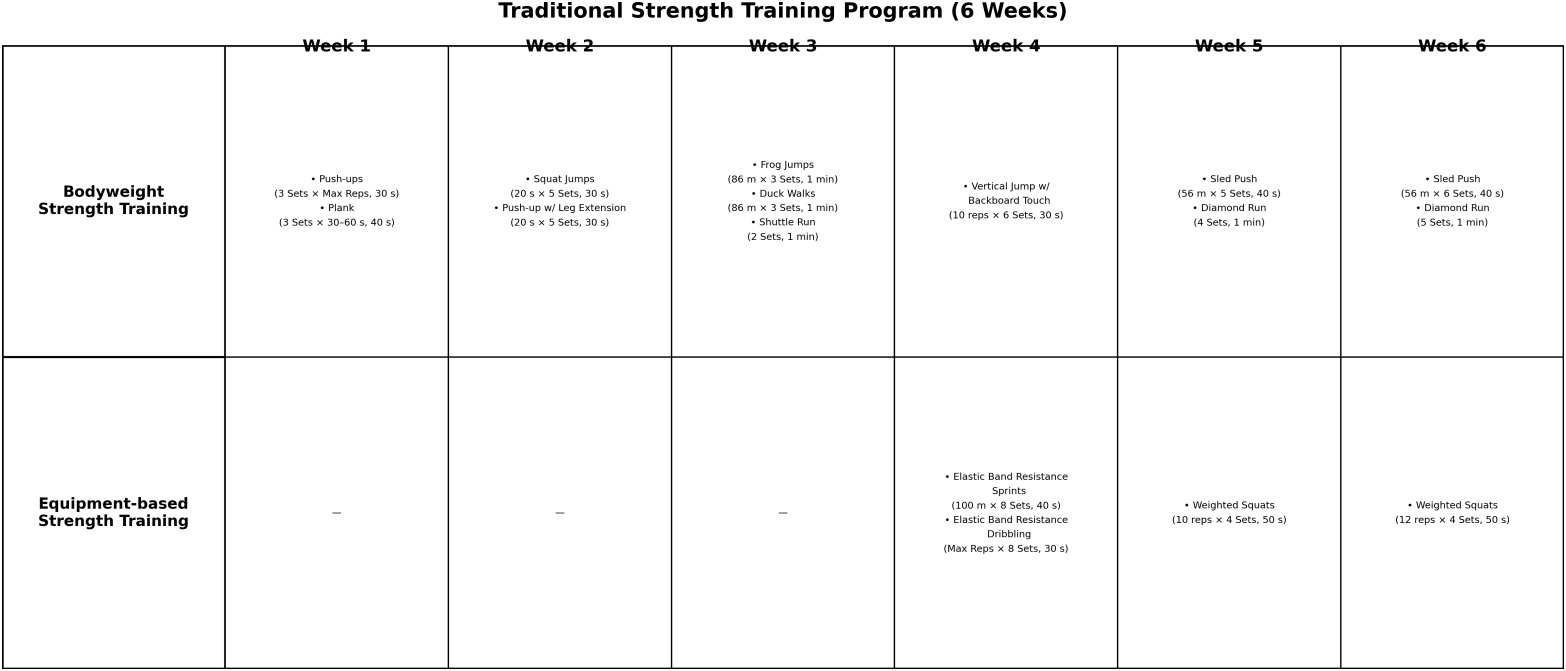
Traditional strength training program (6 Weeks).

The TST group performed only the traditional strength training program and did not receive any additional core stabilization exercises. The traditional strength training program was implemented over six weeks and consisted of progressive bodyweight and equipment-based exercises designed to improve upper- and lower-extremity strength, muscular endurance, explosive power, and basketball-specific movement performance. The program included push-ups, planks, squat jumps, push-ups with leg extension, frog jumps, duck walks, shuttle runs, vertical jumps with backboard touch, elastic band-resisted sprints, elastic band-resisted dribbling, sled push, diamond run, and weighted squats. Details of the traditional strength training program are presented in **Figure 3**.

Each traditional strength training session lasted approximately 45–50 minutes. Training volume increased progressively from three sets per exercise in Week 1 to four to eight sets by Weeks 5–6. Work intervals ranged from 20 seconds to 1 minute depending on the exercise type, while rest intervals between sets ranged from 30 to 60 seconds throughout the intervention. Progression was systematically applied through increases in set number, exercise complexity, and external resistance across the intervention period.

All training sessions were supervised by a qualified physiotherapist to ensure correct exercise execution and participant safety. Participants who reported discomfort were instructed to modify or discontinue the relevant exercise. No adverse events or training-related injuries were reported during the six-week intervention period, suggesting that both intervention protocols were feasible and well tolerated in this adolescent athletic population.

Throughout the six-week intervention period, all participants continued their regular team basketball practice, which consisted of three on-court training sessions per week (approximately 90 minutes each) plus one weekend match, delivered by the participants’ own club coaching staff independently of the study protocol. The CST+TST and TST programs described above were implemented as supplementary sessions on non-overlapping days and were not intended to replace or modify routine basketball training. Adherence to team practice was verbally confirmed with coaches and parents at each weekly study session but was not formally logged with objective monitoring tools (e.g., training-load diaries or wearable sensors), and the volume of concurrent basketball-specific activity was therefore not quantified. Because both groups maintained comparable access to regular team training, between-group differences are unlikely to be explained by differential basketball exposure; however, the absence of quantified monitoring of concurrent sport-specific load is acknowledged as a limitation that should be addressed with objective tracking in future trials.

### 2.5 Outcome measures

All assessments were performed before and after the intervention period. The primary outcome measures were postural alignment, static balance, dynamic balance, and core stability.

#### 2.5.1 Postural alignment assessment

Postural alignment was assessed using the Apecs-AI Posture Evaluation and Correction System^®^ (New Body Technologies SAS, Grenoble, France). Apecs-AI is a digital posture analysis system that uses artificial intelligence-supported algorithms and photogrammetric methods to evaluate body posture from photographs (Moraru et al., 2025; Trovato et al., 2022). Compared with conventional clinical methods, such as plumb-line assessment, or more complex three-dimensional marker-based systems, Apecs-AI provides a rapid, accessible, and non-invasive method for postural screening using mobile devices (Irfan et al., 2024; Trovato et al., 2022). During the assessment, anatomical reference points were identified and photographic images were obtained to evaluate body symmetry and postural alignment. Postural parameters were assessed in the anterior, posterior, and sagittal planes. In the anterior and posterior views, variables such as shoulder alignment, axillary level, pelvic tilt, knee alignment, and trunk asymmetry were evaluated. In the sagittal view, parameters including head inclination, trunk inclination, and foot angle were analyzed (Irfan et al., 2024; Moraru et al., 2025). Previous studies have reported that Apecs-AI demonstrates acceptable to high reliability for several postural parameters. Trovato et al. reported that 13 of 22 tested parameters showed intraclass correlation coefficients above 0.90, indicating high measurement consistency (Trovato et al., 2022). However, lower reliability values have also been reported for specific parameters, such as trunk asymmetry, suggesting that some measurements should be interpreted with caution (Moraru et al., 2025). In the present study, the APECS total posture score was used for statistical analysis, with lower scores indicating better postural alignment.

#### 2.5.2 Static balance assessment

Static balance was assessed using the Balance Error Scoring System (BESS). BESS is a rapid, portable, and field-applicable clinical assessment method commonly used to evaluate postural stability, particularly in athletic populations (Bell et al., 2011; Leong, 2019). Although it was originally developed to identify balance impairments following concussion, it is also widely used for athlete performance monitoring and injury risk assessment (Bell et al., 2011; Long, 2017). The BESS protocol consists of six testing conditions formed by three stance positions performed on two different surfaces. The three stance positions include double-leg stance, single-leg stance, and tandem stance. In the double-leg stance, the participant stands with both feet together. In the single-leg stance, the participant stands on the non-dominant leg. In the tandem stance, the non-dominant foot is positioned behind the dominant foot in a heel-to-toe alignment. Each stance is performed on both a firm surface and a foam surface. During each trial, participants were instructed to keep their eyes closed and hands placed on the iliac crests for 20 seconds. Balance errors were recorded throughout each condition. One point was added for each error, and the maximum error score for each trial was 10. A lower BESS score indicated better static balance performance (Guskiewicz et al., 2001; Leong, 2019). Reported reliability of the total BESS score is moderate, with interrater and intrarater intraclass correlation coefficients (ICC) of 0.57 and 0.74, respectively, and a corresponding minimum detectable change of 9.4 (interrater) and 7.3 (intrarater) points (Finnoff et al., 2009); a subsequent study in healthy adults similarly reported an interrater ICC of 0.66 for the total BESS score, together with moderate criterion validity against force-platform sway measures (Kleffelgaard et al., 2018). This moderate reliability should be considered when interpreting BESS change scores in the present study, and changes below the reported minimum detectable change thresholds were interpreted with appropriate caution.

#### 2.5.3 Dynamic balance assessment

Dynamic balance was assessed using the Y Balance Test (YBT). The YBT is a standardized field-based test used to evaluate dynamic balance, functional motor control, and injury risk. It is considered a modernized and standardized version of the Star Excursion Balance Test and has been widely used in athletic populations (Mohammadi et al., 2012; Nelson et al., 2021; Plisky et al., 2021). A systematic review and meta-analysis pooling the available reliability literature reported good-to-excellent interrater and intrarater reliability for individual reach directions and the composite reach score, with intraclass correlation coefficients generally ranging from approximately 0.85 to 1.00 across studies; the same review also supported the discriminant and predictive validity of the YBT-LQ, with a composite reach score below 94% and an anterior reach asymmetry greater than 4 cm both associated with increased lower-extremity injury risk in athletic populations (Plisky et al., 2021).

During the test, the participant stood barefoot on one leg on a central platform and attempted to reach as far as possible with the free limb in three directions: anterior, posteromedial, and posterolateral. Throughout the test, participants were instructed to maintain balance, keep their hands on their hips, and avoid using the reaching foot for support during the reach movement (Mohammadi et al., 2017; Nelson et al., 2021). Higher YBT scores indicated better dynamic balance performance (Butler et al., 2013; Nelson et al., 2021).

#### 2.5.4 Core stability assessment

Core stability-related performance was assessed using the Sport-Specific Core Muscle Strength and Stability Plank Test. This field-based test is designed to evaluate static core endurance and dynamic lumbopelvic control under conditions requiring sequential upper- and lower-extremity movements. Unlike conventional static plank endurance tests, this protocol challenges the athlete’s ability to maintain trunk alignment while limb position and loading demands change progressively (Taylor et al., 2017).

The test was performed in a standard prone plank position, with participants supported on their forearms and toes. The protocol consisted of eight consecutive stages performed without rest. First, participants maintained the standard prone plank position for 60 seconds. This was followed by seven progressive stages: right arm elevation for 15 seconds, left arm elevation for 15 seconds, right leg elevation for 15 seconds, left leg elevation for 15 seconds, simultaneous elevation of the right arm and left leg for 15 seconds, simultaneous elevation of the left arm and right leg for 15 seconds, and a final return to the standard prone plank position for 30 seconds. One complete cycle lasted 180 seconds. If a participant successfully completed the full cycle, the sequence was repeated until fatigue or failure to maintain the required position occurred.

Test performance was recorded as the total time, in seconds, during which the participant maintained correct technique. Throughout the test, participants were instructed to maintain a straight body line from the shoulders through the hips and ankles. The test was terminated if the participant demonstrated loss of alignment, including excessive hip elevation or dropping, excessive trunk rotation, contact of any body part with the floor other than the forearms and feet, or inability to correct posture despite verbal instruction (Aggarwal et al., 2010; Taylor et al., 2017). A longer test duration indicated better core endurance and lumbopelvic stability performance (Boccolini et al., 2013).

This assessment was selected because it provides a practical, sport-relevant measure of trunk control under progressive limb perturbations relevant to basketball-specific actions, and is feasible in field-based youth sport settings. This progressive, multi-stage plank protocol is consistent with the sport-specific endurance plank test described by Tong et al., who reported excellent test-retest reliability (ICC = 0.99, 95% CI 0.98–0.99) and low measurement error (coefficient of variation = 2.0 ± 1.56%) following a single familiarization trial, together with construct validity demonstrated through a greater than 50% increase in trunk muscle electromyographic activation during the test and a clear discrimination of an approximately 30% reduction in core endurance following a pre-fatigue protocol (Tong et al., 2014). Consistent with this recommendation, no separate familiarization trial was administered prior to the pre-test assessment in the present study; participants therefore performed the multi-stage protocol for the first time at baseline, which is considered further as a potential source of practice-related improvement in the Discussion. Nevertheless, core function is multidimensional and cannot be fully captured by a single plank-based test; future studies should complement this assessment with objective measures such as isokinetic trunk strength testing.

### 2.6 Statistical analysis

Statistical analyses were performed using IBM SPSS Statistics for Windows, Version 26.0 (IBM Corp., Armonk, NY, USA). Continuous variables were presented as mean ± standard deviation (SD), while categorical variables were reported as frequency and percentage where applicable.

The normality of outcome variables was assessed using visual inspection and the Shapiro–Wilk test, given the small sample size. Homogeneity of variance was examined using Levene’s test. Most variables met the normality assumption, except for the CST+TST post-test values of the Plank Test and APECS Total score. However, as group sizes were equal and Levene’s tests indicated no significant violations of homogeneity of variance, a parametric approach was retained. Baseline characteristics, including age, height, and body mass, were compared between groups using independent samples t-tests.

Intervention effects were analyzed using a 2 × 2 mixed-design analysis of variance (ANOVA), with Group (CST+TST vs. TST) as the between-subject factor and Time (pre-test vs. post-test) as the within-subject factor. The Group × Time interaction was considered the primary indicator of differential intervention effect. Partial eta squared (ηp²) was reported as the effect size for ANOVA and interpreted as small (≥0.01), medium (≥0.06), or large (≥0.14). When a significant Group × Time interaction was identified, within-group simple-effects comparisons were conducted using paired-samples t-tests to characterize pre–post changes within each group. These paired t-tests represent simple-effects decomposition of the significant interaction rather than multiplicity-corrected *post-hoc* tests; given the small number of planned, theory-driven comparisons (one within-group test per group per outcome), no additional correction (e.g., Bonferroni) was applied to these simple-effects tests. Hedges’ g was calculated to estimate within-group effect sizes, accounting for the small sample size.

All statistical tests were two-tailed, and statistical significance was set at p < 0.05. Exact p-values were reported where appropriate. Findings other than the primary BESS interaction effect were interpreted cautiously, particularly where significance would not remain under conservative correction procedures.

## 3 Results

### 3.1 Baseline characteristics of the groups

The baseline characteristics of the participants are presented in **Table 1**. Independent samples t-tests showed no statistically significant differences between the core stabilization plus traditional strength training group and the traditional strength training group in age, height, or body mass (p>0.05). Pre-test scores for all primary and secondary outcome measures were also compared between groups using independent samples t-tests. No statistically significant between-group differences were found at baseline for BESS (t(22) = -0.40, p = .690), YBT-Right (t(22) = 1.45, p = .162), YBT-Left (t(22) = 1.13, p = .269), the plank test (t(22) = 0.64, p = .526), or the APECS total posture score (t(22) = -0.12, p = .908), confirming group equivalence on the outcome variables themselves prior to the intervention.

### 3.2 Comparison of pre-test and post-test BESS scores

**Figure 4:**
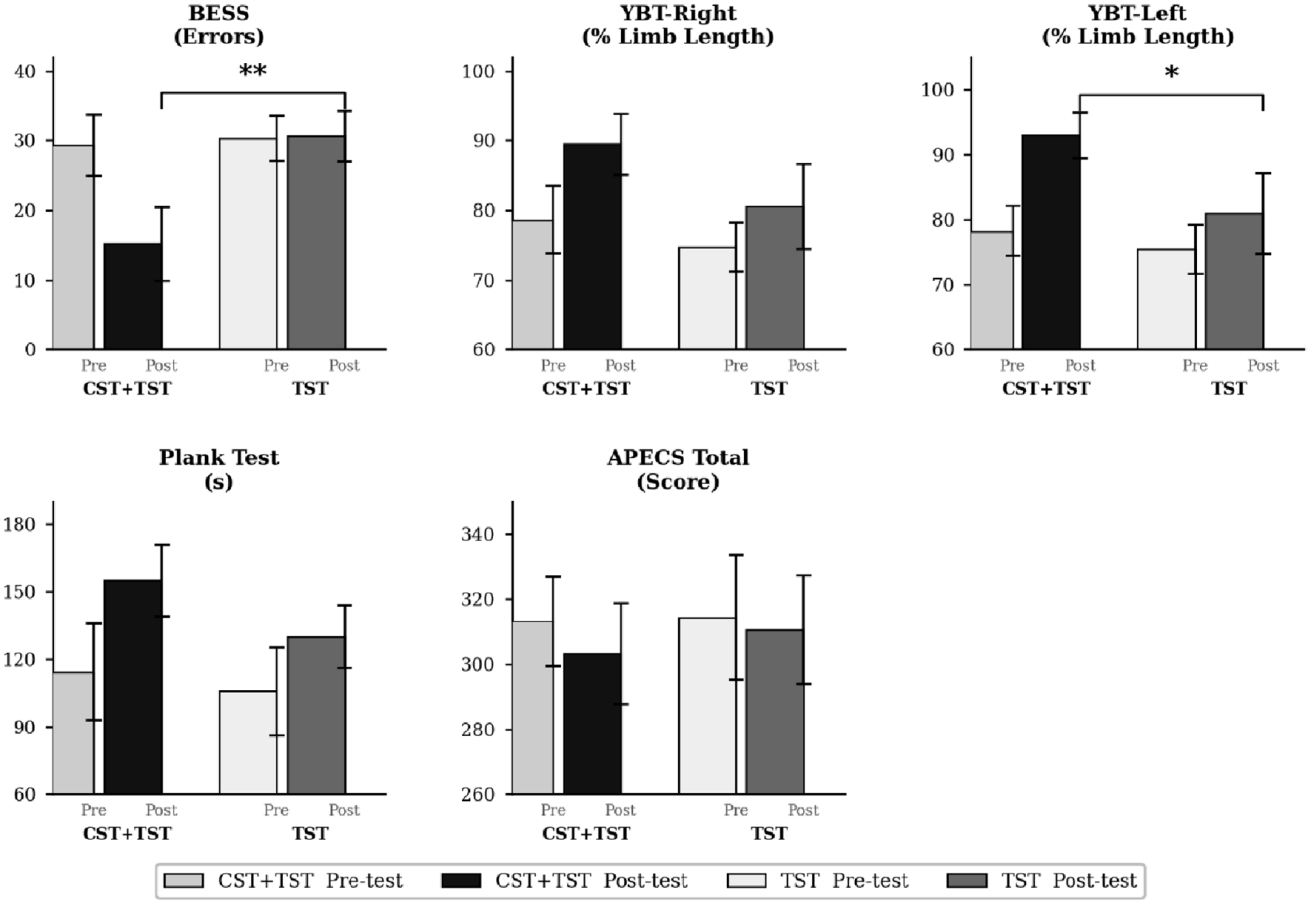
Mean ± 95% confidence interval for each primary outcome at pre-test and post-test in the CST+TST and TST groups. Error bars represent 95% CI. **Group×Time interaction p <.001; *p = .022 (2×2 mixed-design ANOVA, df = 1,22). For BESS and APECS Total, lower scores indicate better performance. For YBT-Right, YBT-Left, and plank test, higher scores indicate better performance. BESS, Balance error scoring system; YBT, Y balance test; APECS, Apecs-AI posture evaluation and correction system; CST+TST, Core stabilization plus traditional strength training; TST, Traditional strength training only.

The mixed ANOVA results are shown in **Table 2**, and pre-test and post-test scores for all primary outcomes are illustrated in **Figure 4**. *Post-hoc* within-group analyses (**Table 3**) showed that BESS scores decreased significantly in the CST+TST group (t = 5.41, p <.001, Hedges’ g = 1.45, 95% CI for change [−19.93, −8.41] errors), indicating improved static balance. No significant within-group change was observed in the TST group (t = −0.34, p = .744, 95% CI [−1.86, +2.52] errors).

**Table 2:** Results of the 2×2 mixed-design ANOVA: Group, Time, and Group×Time interaction effects.

Variable	Effect	F(1,22)	p	ηp²
BESS	Group	12.757	.002	.367
	Time	24.421	<.001	.526
	Group×Time	26.832	<.001	.549
YBT-Right (%)	Group	6.801	.016	.236
	Time	21.055	<.001	.489
	Group×Time	1.907	.181	.080
YBT-Left (%)	Group	11.390	.003	.341
	Time	29.253	<.001	.571
	Group×Time	6.099	.022	.217
Plank Test (s)	Group	3.064	.094	.122
	Time	25.589	<.001	.538
	Group×Time	1.649	.212	.070
APECS Total	Group	0.206	.654	.009
	Time	2.025	.169	.084
	Group×Time	0.381	.543	.017

Values are presented as mean ± standard deviation. F values are from the 2×2 mixed-design ANOVA. ηp² = partial eta squared. df = (1, 22) for all effects. Bold p-values indicate significant Group×Time interactions (p <.05). CST+TST, Core Stabilization plus Traditional Strength Training; TST, Traditional Strength Training only; BESS, Balance Error Scoring System; YBT, Y Balance Test; APECS, Apecs-AI Posture Evaluation and Correction System.

**Table 3:** Within-group simple effects: pre-test and post-test scores.

Variable	Group	Pre-test	Post-test	t	p	Hedges’ g
BESS	CST+TST	29.33 ± 6.91	15.17 ± 8.31	5.414	<.001	-1.45
	TST	30.33 ± 5.05	30.67 ± 5.73	-0.335	.744	0.09
YBT-Right (%)	CST+TST	78.60 ± 7.58	89.51 ± 6.83	-7.414	<.001	1.99
	TST	74.68 ± 5.52	80.53 ± 9.61	-1.752	.108	0.47
YBT-Left (%)	CST+TST	78.22 ± 6.05	92.92 ± 5.49	-12.045	<.001	3.23
	TST	75.43 ± 6.00	80.92 ± 9.81	-1.555	.148	0.42
Plank Test (s)	CST+TST	114.25 ± 33.69	155.00 ± 24.95	-4.101	.002	1.10
	TST	105.75 ± 30.89	130.00 ± 21.83	-2.976	.013	0.80
APECS Total	CST+TST	313.17 ± 21.50	303.25 ± 24.46	1.417	.184	-0.38
	TST	314.42 ± 30.32	310.50 ± 26.32	0.580	.573	-0.16

Values are presented as mean ± standard deviation. *Post-hoc* paired samples t-tests were conducted within each group following significant Group×Time interactions in the mixed ANOVA. Hedges’ g was calculated using paired difference SD to account for small sample size. Statistically significant p-values (p <.05) are indicated in bold. Percentage changes from pre-test: BESS: CST+TST −48.3%, TST + 1.1%; YBT-Right: CST+TST +13.9%, TST + 7.8%; YBT-Left: CST+TST +18.8%, TST + 7.3%; Plank: CST+TST +35.7%, TST + 22.9%; APECS: CST+TST −3.2%, TST −1.2%. CST+TST, Core Stabilization plus Traditional Strength Training; TST, Traditional Strength Training only; BESS, Balance Error Scoring System; YBT, Y Balance Test; APECS, Apecs-AI Posture Evaluation and Correction Systeym.

The Group×Time interaction for BESS was statistically significant (F(1,22) = 26.83, p <.001, ηp² = .549, **Table 2**), indicating that the CST+TST group improved significantly more than the TST group in static balance. The large effect size for BESS (ηp² = .549) and the CST+TST group’s mean improvement of 48.3% from baseline represent a clinically relevant gain in static balance performance.

### 3.3 Comparison of pre-test and post-test Y balance test scores

The mixed ANOVA results for Y Balance Test are presented in **Table 2**. *Post-hoc* within-group analyses (**Table 3**) showed that both right-side (95% CI [+7.67, +14.14]%) and left-side (95% CI [+12.01, +17.38]%) YBT scores increased significantly in the CST+TST group (p <.001). In the TST group, YBT scores increased numerically on both sides (right: 95% CI [−1.50, +13.22]%; left: 95% CI [−2.28, +13.24]%); however, these changes were not statistically significant (p >.05).

The Group×Time interaction results are shown in **Table 2**, with *post-hoc* comparisons in **Table 3**. The Group×Time interaction was not significant for YBT-Right (F(1,22) = 1.91, p = .181, ηp² = .080). However, a significant Group×Time interaction was observed for YBT-Left (F(1,22) = 6.10, p = .022, ηp² = .217), indicating greater improvement in the CST+TST group. These findings suggest that the addition of core stabilization exercises contributed to greater improvement in selected dynamic balance outcomes. The large effect size for YBT-Left (ηp² = .217, medium-to-large) and the CST+TST group’s mean improvement of +14.7% represent a clinically relevant gain in dynamic balance asymmetry relevant to injury risk screening thresholds in basketball players.

### 3.4 Comparison of pre-test and post-test plank test scores

The mixed ANOVA results for the plank test are shown in **Table 2**. *Post-hoc* within-group analyses (**Table 3**) showed that plank test performance increased significantly in both the CST+TST group (t = −4.10, p = .002, Hedges’ g = 1.10, 95% CI [+18.88, +62.62] s) and the TST group (t = −2.98, p = .013, Hedges’ g = 0.80, 95% CI [+6.32, +42.18] s).

The Group×Time interaction for the plank test was not significant (F(1,22) = 1.65, p = .212, ηp² = .070). Although both groups showed significant within-group improvements (**Table 3**), the addition of core stabilization training did not produce statistically superior improvements compared with traditional strength training alone.

### 3.5 Comparison of pre-test and post-test APECS total posture scores

The mixed ANOVA results for APECS total posture score are presented in **Table 2**. *Post-hoc* within-group analyses (**Table 3**) showed no statistically significant pre-test to post-test change in either the CST+TST group (t = 1.42, p = .184) or the TST group (t = 0.58, p = .573).

The Group×Time interaction for APECS total posture score is shown in **Table 2**. The Group×Time interaction was not significant (F(1,22) = 0.38, p = .543, ηp² = .017), indicating that neither training approach produced a differential effect on overall postural alignment during the intervention period.

### 3.6 Summary of between-group comparisons

The pre-test and post-test scores for all outcomes are presented graphically in **Figure 4**. As shown in **Table 2**, significant Group×Time interactions were found for BESS (F(1,22) = 26.83, p <.001) and YBT-Left (F(1,22) = 6.10, p = .022) in favor of the CST+TST group. It should be noted that this p-value (p = .022) derives from the revised mixed-design ANOVA and supersedes the value of p = .027 reported in the original analysis, which was based on an independent samples t-test of change scores. No adjustment for multiple comparisons was applied across the five outcomes; applying a Bonferroni correction (α = 0.01) would render the YBT-Left interaction non-significant, and readers should interpret this finding accordingly as an exploratory, hypothesis-generating result. No significant interactions were observed for YBT-Right, plank test, or APECS total posture score. Overall, these findings indicate that adding core stabilization exercises to traditional strength training was more effective for improving static balance and selected dynamic balance outcomes, but not for plank performance or postural alignment.

## 4 Discussion

The present study examined whether adding a six-week core stabilization program to traditional strength training improves postural alignment, static balance, dynamic balance, and core endurance in adolescent male basketball players. The CST+TST group showed superior improvements in static balance (BESS) and left-side dynamic balance (YBT), whereas plank performance improved similarly in both groups and APECS posture scores did not change. These findings suggest that core stabilization training confers balance-specific benefits, while short-term gains in trunk endurance and static alignment are not specifically attributable to the added core component.

### 4.1 Static balance

The most pronounced finding was the significant improvement in static balance in the CST+TST group, consistent with previous evidence that core stabilization training improves postural control in athletic populations (Bell et al., 2011; Reed et al., 2012; Zemková and Zapletalová, 2022). Mechanistically, this may reflect earlier recruitment of deep trunk stabilizers such as the transversus abdominis and multifidus, which support anticipatory spinal stabilization and reduce reliance on slower feedback-mediated corrections (Hodges and Richardson, 1998; Hsu et al., 2018), as well as reduced compensatory trunk and hip motion during single-leg stance, allowing more efficient ankle-strategy control (Yalfani et al., 2023). The large effect size (ηp² = .549) should be interpreted cautiously given the small sample, but its biological plausibility and the consistency of the *post-hoc* findings support its robustness; the mean reduction of 14.2 BESS errors also exceeds the minimal detectable change of 6–10 errors reported in sub-elite athletes, suggesting a clinically meaningful rather than measurement-error-driven change (Finnoff et al., 2009; Kleffelgaard et al., 2018; Mulligan et al., 2013).

### 4.2 Dynamic balance

Dynamic balance also improved in the CST+TST group, consistent with evidence that core training benefits dynamic postural control and lower-extremity function (Abdelraouf et al., 2020; Genc et al., 2019; Çakır and Ergin, 2022), including basketball-specific literature reporting greater dynamic-balance gains from core-stability training than from traditional strength training alone (Dogan and Savaş, 2021; Gong et al., 2024). The 14.7% improvement in left-side YBT performance exceeded the minimal detectable change reported for adolescent athletes (4.90–16.10%), suggesting a real performance change (Myer et al., 2005). However, the between-group difference was significant only for the left side; this asymmetry may reflect limb dominance or side-dependent neuromuscular adaptations, but limb dominance was not recorded, and the YBT-left interaction should be considered exploratory and hypothesis-generating given the absence of correction for multiple comparisons.

### 4.3 Core endurance

Plank test performance improved significantly in both groups, with no significant Group×Time interaction, suggesting that the trunk-endurance gain was not specific to the added core stabilization component; the comparable improvement in the TST group indicates that traditional strength training alone may already be sufficient to improve trunk endurance over six weeks. Part of this within-group improvement may also reflect a test-familiarity, or practice, effect rather than a purely physiological gain: as noted in the Methods, no separate familiarization trial preceded the pre-test assessment, so participants encountered the complex, multi-stage plank protocol for the first time at baseline. Post-test scores may therefore partly reflect improved task coordination, pacing strategy, and procedural understanding of the test itself, rather than a true increase in core endurance, consistent with reliability evidence indicating that a familiarization trial is recommended before this protocol yields stable performance scores (Tong et al., 2014). This is distinct from, and may have occurred in addition to, genuine growth-related neuromuscular development around peak height velocity, which can also increase trunk endurance naturally during adolescence independent of any targeted training (Borato et al., 2025; Williams et al., 2021; Bayrakdar et al., 2020; Ozmen et al., 2020; da Silva Sartori et al., 2022). Future studies should incorporate a standardized familiarization trial alongside maturation indicators such as Tanner staging to better distinguish learning-related, developmental, and intervention-specific effects.

### 4.4 Postural alignment

No significant improvement was observed in the APECS total posture score, indicating a dissociation between functional balance improvement and static postural alignment. Static balance (BESS) reflects short-term neuromuscular control and sensory integration that may respond quickly to training (Borghuis et al., 2008), whereas static alignment reflects more chronic structural determinants such as growth, joint mobility, and habitual posture (Liang et al., 2025). A six-week program, and a photogrammetric measure such as APECS, may therefore be insufficiently sensitive to detect early structural change. Meaningful alignment changes may instead require longer interventions or more targeted corrective exercise content (Liang et al., 2025; Luo et al., 2023).

### 4.5 Practical implications

From a practical standpoint, the clinically meaningful BESS improvement suggests that a six-week core stabilization program (3×/week) can be integrated as a balance-oriented adjunct to traditional strength training for adolescent basketball players (Myer et al., 2005), particularly during pre-season or developmental phases when athletes may experience transient coordination and balance deficits associated with rapid growth. Because plank performance did not differ between groups, traditional strength training alone may suffice for general trunk endurance, while core stabilization exercises may be more valuable for static postural control (Gál-Pottyondy et al., 2025; Hung et al., 2019); the program may therefore be positioned as a short-term, balance-focused microcycle supporting movement control during landing, pivoting, cutting, and defensive actions (Cowley et al., 2006; Pfile et al., 2016). It is important to emphasize that these findings describe the additive value of core stabilization training when layered onto an existing traditional strength training program, not the effect of core stabilization training in isolation. Because resistance training is already a standard component of preparation for competitive youth basketball players in this age range (Faigenbaum et al., 2009; Gonzalo-Skok et al., 2025), the practical implication is that coaches and practitioners should incorporate core stabilization exercises alongside, rather than instead of, the strength training their athletes are already performing; the present results do not support replacing traditional strength training with a core-stabilization-only approach.

### 4.6 Limitations

Several limitations should be acknowledged. The sample size was small, with adequate *post-hoc* power only for the primary BESS outcome, so findings for plank performance, postural alignment, right-side dynamic balance, and the side-specific YBT response warrant cautious interpretation. Biological maturation and limb dominance were not assessed, limiting control for maturity-related variability and interpretation of the side-specific YBT finding. Participant blinding was not feasible given the nature of the intervention, although assessors were blinded to reduce observer bias. Because the CST+TST group received an additional training component, observed balance improvements may partly reflect greater total training volume rather than the isolated effect of core stabilization content. Finally, the six-week duration may have been insufficient to detect changes in static postural alignment, and the YBT-left result should be regarded as exploratory given the absence of correction for multiple comparisons.

### 4.7 Future directions

Future studies should use larger, *a priori* powered samples, assess biological maturation and limb dominance, and apply volume-matched control conditions. Longer interventions with follow-up assessments are needed to determine whether balance improvements persist and whether extended programs affect postural alignment, and future work should examine whether gains in clinical balance tests translate into sport-specific outcomes such as landing mechanics, change-of-direction performance, injury incidence, and skill execution.

### 4.8 Conclusion

In conclusion, adding a core stabilization exercise program to traditional strength training improved static balance and selected dynamic balance outcomes in adolescent male basketball players. However, the combined program did not produce superior improvements in plank test performance or APECS total posture score compared with traditional strength training alone. Because traditional strength training is already a widely implemented component of preparation for competitive youth basketball players in this age group, these findings should be interpreted as evidence for the additive benefit of incorporating core stabilization exercises into an existing strength training program, rather than as support for core stabilization training as a standalone alternative. These findings suggest that core stabilization exercises, when added to traditional strength training, may serve as an effective complementary strategy for enhancing balance performance in young basketball players, while improvements in trunk endurance and static postural alignment may require either longer interventions, more specific corrective exercise content, or maturation-controlled study designs.

## Data Availability

The data analyzed in this study is subject to the following licenses/restrictions: The dataset generated and analyzed during the current study is not publicly available due to the inclusion of data obtained from minor participants and the need to protect participant privacy and confidentiality. Access to the dataset is restricted in accordance with ethical requirements and parental consent conditions. De-identified data may be made available from the corresponding author upon reasonable request, subject to institutional approval and applicable ethical regulations. Requests to access these datasets should be directed to dazim@bandirma.edu.tr.

## References

[B1] Abdelraouf O. R. Abdel-aziem A. A. Selim A. O. Ali O. I. (2020). Effects of core stability exercise combined with virtual reality in collegiate athletes with nonspecific low back pain: a randomized clinical trial. Bull. Faculty Phys. Ther. 25, 7. doi: 10.1186/s43161-020-00003-x 38164791

[B2] Aggarwal A. Kumar S. Kalpana Z. Jitender M. Sharma V. (2010). The relationship between core stability performance and the lower extremities static balance performance in recreationally active individuals. Nigerian J. Med. Rehabil. 15 (23), 11–16. doi: 10.34058/njmr.v15i1.2.52

[B3] Amato A. Cortis C. Tropea M. Politi M. Fusco A. Musumeci G. (2025). Comparison of the core training and mobility training effects on basketball athletic performance in young players: a comparative experimental study. Sports 13, 398. doi: 10.3390/sports13110398 41295781 PMC12655985

[B4] Barrio E. D. Ramirez-Campillo R. Garcia De Alcaraz Serrano A. Hernandez-García R. (2022). Effects of core training on dynamic balance stability: a systematic review and meta-analysis. J. Sports Sci. 40, 1815–1823. doi: 10.1080/02640414.2022.2110203 35976032

[B5] Bayrakdar A. Boz H. K. Işıldar Ö. (2020). The investigation of the effect of static and dynamic core training on performance on football players. Turkish J. Sport Exercise 22, 87–95.

[B6] Bell D. R. Guskiewicz K. M. Clark M. A. Padua D. A. (2011). Systematic review of the balance error scoring system. Sports Health 3, 287–295. doi: 10.1177/1941738111403122 23016020 PMC3445164

[B7] Boccolini G. Brazzit A. Bonfanti L. Alberti G. (2013). Using balance training to improve the performance of youth basketball players. Sport Sci. Health 9, 37–42. doi: 10.1007/s11332-013-0143-z 23956797 PMC3713268

[B8] Borato L. A. Whatman C. Walters S. Read P. (2025). What do we know (and not know) about adolescent awkwardness in youth sports? a narrative review. Int. J. Sports Sci. Coaching 20, 2257–2267. doi: 10.1177/17479541251364101

[B9] Borghuis J. Hof A. L. Lemmink K. A. (2008). The importance of sensory-motor control in providing core stability: implications for measurement and training. Sports Med. 38, 893–916. doi: 10.2165/00007256-200838110-00002 18937521

[B10] Butler R. J. Lehr M. E. Fink M. L. Kiesel K. B. Plisky P. J. (2013). Dynamic balance performance and noncontact lower extremity injury in college football players: an initial study. Sports Health 5, 417–422. doi: 10.1177/1941738113498703 24427412 PMC3752196

[B11] Çakır M. Ergin E. (2022). The effect of core training on agility, explosive strength and balance in young female volleyball players. J. Sport Sci. Res. 7, 525–535.

[B12] Cowley H. R. Ford K. R. Myer G. D. Kernozek T. W. Hewett T. E. (2006). Differences in neuromuscular strategies between landing and cutting tasks in female basketball and soccer athletes. J. Athletic Training 41, 67. PMC1421490 16619097

[B13] da Silva Sartori C. B. Montagner P. C. Borin J. P. (2022). Relación entre la estabilidad del core y el equilibrio postural en las habilidades biomotoras de los jóvenes atletas de baloncesto: una revisión sistemática. Retos: Nuevas Tendencias en Educación Física Deporte Y. Recreación 44 (2022), 749–755.

[B14] Dogan O. Savaş S. (2021). Effect of an 8-weeks core training program applied to 12-14 years old basketball players on strength, balance and basketball skill. Pakistan J. Med. Health Sci. 15.

[B15] Dong K. Yu T. Chun B. (2023). Effects of core training on sport-specific performance of athletes: a meta-analysis of randomized controlled trials. Behav. Sci. 13, 148. doi: 10.3390/bs13020148 36829378 PMC9952339

[B16] Faigenbaum A. D. Kraemer W. J. Blimkie C. J. R. Jeffreys I. Micheli L. J. Nitka M. . (2009). Youth resistance training: updated position statement paper from the National Strength and Conditioning Association. J. Strength Conditioning Res. 23, S60–S79. doi: 10.1519/jsc.0b013e31819df407 19620931

[B17] Finnoff J. T. Peterson V. J. Hollman J. H. Smith J. (2009). Intrarater and interrater reliability of the balance error scoring system (BESS). PM&R 1, 50–54. 19627872 10.1016/j.pmrj.2008.06.002

[B18] Fransen J. Deprez D. Pion J. Tallir I. B. D’Hondt E. Vaeyens R. . (2014). Changes in physical fitness and sports participation among children with different levels of motor competence: a 2-year longitudinal study. Pediatr. Exercise Sci. 26, 11–21. doi: 10.1123/pes.2013-0005 24018944

[B19] Gál-Pottyondy A. Pálya Z. Trzaskoma L. Kiss R. M. (2025). Integrating trunk endurance, dynamic stability, and in-game performance analysis in youth elite basketball players. BMC Sports Sci. Med. Rehabil. 17, 269. doi: 10.1186/s13102-025-01285-1 40993779 PMC12462323

[B20] Genc H. Cigerci A. Sever O. (2019). Effect of 8-week core training exercises on physical and physiological parameters of female handball players. Phys. Educ. Students 23, 297–305. doi: 10.15561/20755279.2019.0604

[B21] Gong J. Gao H. Sui J. Qi F. (2024). The effect of core stability training on the balance ability of young male basketball players. Front. Physiol. 14, 1305651. doi: 10.21203/rs.3.rs-3295879/v1 38250660 PMC10796723

[B22] Gonzalo-Skok O. Arede J. Dos’Santos T. (2025). Effects of strength training and detraining considering maturity status in youth highly trained basketball players. PloS One 20, e0317879. doi: 10.1371/journal.pone.0317879 39937825 PMC11819605

[B23] Guskiewicz K. M. Ross S. E. Marshall S. W. (2001). Postural stability and neuropsychological deficits after concussion in collegiate athletes. J. Athletic Training 36, 263. PMC155417 12937495

[B24] Hodges P. W. Richardson C. A. (1998). Delayed postural contraction of transversus abdominis in low back pain associated with movement of the lower limb. Clin. Spine Surg. 11, 46–56. doi: 10.1097/00002517-199802000-00008 9493770

[B25] Hsu S.-L. Oda H. Shirahata S. Watanabe M. Sasaki M. (2018). Effects of core strength training on core stability. J. Phys. Ther. Sci. 30, 1014–1018. doi: 10.1589/jpts.30.1014 30154592 PMC6110226

[B26] Hung K. C. Chung H. W. Yu C. C. W. Lai H. C. Sun F. H. (2019). Effects of 8-week core training on core endurance and running economy. PloS One 14, e0213158. doi: 10.1371/journal.pone.0213158 30849105 PMC6407754

[B27] Irfan U. Asif S. Mumtaz M. Jamal S. Khalid F. Fatima K. . (2024). Prevalence of poor body posture among physiotherapists using APECS. J. Health Rehabil. Res. 4, 1323–1327. doi: 10.61919/jhrr.v4i1.602

[B28] Kleffelgaard I. Langhammer B. Sandhaug M. Pripp A. H. Søberg H. L. (2018). Measurement properties of the modified and total balance error scoring system–the BESS, in a healthy adult sample. Eur. J. Physiotherapy 20, 25–31. doi: 10.1080/21679169.2017.1352020 37339054

[B29] Leong D. (2019). Sports-related traumatic brain injury: screening and management. Sports Health Exercise Med. doi: 10.5772/intechopen.88442

[B30] Li Y. (2023). Strengthening the abdominal core on balance in basketball players. Rev. Bras. Med. do Esporte 29, e2022_0598. doi: 10.1590/1517-8692202329012022_0598

[B31] Liang I.-J. Lin L. L. Huang C.-C. (2025). The effects of different core stability training on trunk stability and athletic performance in adolescent female basketball players. J. Sport Rehabil. 34, 747–753. doi: 10.1123/jsr.2024-0037 40010356

[B32] Long M. (2017). The Effects of a Six Week Lumbopelvic Control and Balance Training Program in High School Basketball Players (Morgantown: West Virginia University).

[B33] Luo S. Soh K. G. Zhao Y. Soh K. L. Sun H. Nasiruddin N. J. M. . (2023). Effect of core training on athletic and skill performance of basketball players: a systematic review. PloS One 18, e0287379. doi: 10.1371/journal.pone.0287379 37347733 PMC10286970

[B34] Mohammadi V. Alizadeh M. Gaieni A. (2012). The effects of six weeks strength exercises on static and dynamic balance of young male athletes. Procedia-Social Behav. Sci. 31, 247–250. doi: 10.1016/j.sbspro.2011.12.050 38826717

[B35] Mohammadi V. Hilfiker R. Jafarnezhadgero A. A. Jamialahmadi S. Karimizadeh Ardakani M. Granacher U. (2017). Relationship between training-induced changes in the star excursion balance test and the Y balance test in young male athletes. Ann. Appl. Sport Sci. 5, 31–38. doi: 10.29252/acadpub.aassjournal.5.3.31

[B36] Moraru L. Badau A. Litoi F. M. Petrescu A. M. Manescu C. O. Smidu N. (2025). Identification of postural deviations and trunk asymmetries in physical therapy students using an AI-based mobile application. Balneo PRM Res. J. 16. doi: 10.12680/balneo.2025.802

[B37] Mueller S. Mueller J. Stoll J. Mayer F. (2022). Effect of six-week resistance and sensorimotor training on trunk strength and stability in elite adolescent athletes: a randomized controlled pilot trial. Front. Physiol. 13, 802315. doi: 10.3389/fphys.2022.802315 35370766 PMC8969222

[B38] Mulligan I. J. Boland M. A. McIlhenny C. V. (2013). The balance error scoring system learned response among young adults. Sports Health 5, 22–26. doi: 10.1177/1941738112467755 24381697 PMC3548663

[B39] Myer G. D. Ford K. R. Palumbo O. P. Hewett T. E. (2005). Neuromuscular training improves performance and lower-extremity biomechanics in female athletes. J. Strength Conditioning Res. 19, 51–60. doi: 10.1519/00124278-200502000-00010 15705045

[B40] Nelson S. Wilson C. S. Becker J. (2021). Kinematic and kinetic predictors of Y-balance test performance. Int. J. Sports Phys. Ther. 16, 371. doi: 10.26603/001c.21492 33842033 PMC8016412

[B41] Ozmen T. Aydogmus M. Yana M. Simsek A. (2020). Effect of core strength training on balance, vertical jump height and throwing velocity in adolescent male handball players. J. Sports Med. Phys. Fitness 60, 693–699. doi: 10.23736/s0022-4707.20.10382-7 32162502

[B42] Pan J. Wei M. (2022). Scientific physical core strength training of adolescent group. Rev. Bras. Med. do Esporte 28, 235–237. doi: 10.1590/1517-8692202228032021_0470

[B43] Pfile K. R. Gribble P. A. Buskirk G. E. Meserth S. M. Pietrosimone B. G. (2016). Sustained improvements in dynamic balance and landing mechanics after a 6-week neuromuscular training program in college women’s basketball players. J. Sport Rehabil. 25, 233–240. doi: 10.1123/jsr.2014-0323 26355913

[B44] Plisky P. Schwartkopf-Phifer K. Huebner B. Garner M. B. Bullock G. (2021). Systematic review and meta-analysis of the Y-balance test lower quarter: reliability, discriminant validity, and predictive validity. Int. J. Sports Phys. Ther. 16, 1190. 34631241 10.26603/001c.27634 PMC8486397

[B45] Quatman-Yates C. C. Quatman C. E. Meszaros A. J. Paterno M. V. Hewett T. E. (2012). A systematic review of sensorimotor function during adolescence: a developmental stage of increased motor awkwardness? Br. J. Sports Med. 46, 649–655. doi: 10.1136/bjsm.2010.079616 21459874 PMC4157222

[B46] Reed C. A. Ford K. R. Myer G. D. Hewett T. E. (2012). The effects of isolated and integrated ‘core stability’ training on athletic performance measures: a systematic review. Sports Med. 42, 697–706. 22784233 10.2165/11633450-000000000-00000 PMC4166601

[B47] Rodríguez-Perea Á. Reyes-Ferrada W. Jerez-Mayorga D. Chirosa Ríos L. J. Van Den Tillar R. Chirosa Ríos I. . (2023). Core training and performance: a systematic review with meta-analysis. Biol. Sport 40, 975–986. doi: 10.5114/biolsport.2023.123319 37867742 PMC10588579

[B48] Schulte S. Lukas M. Bopp J. Zschorlich V. Büsch D. (2025). Relation between core strength, core stability, and athletic performance—a mediation analysis approach. Front. Sports Active Living 7, 1669023. 10.3389/fspor.2025.1669023 PMC12698640 41393317

[B49] Taylor J. B. Nguyen A.-D. Paterno M. V. Huang B. Ford K. R. (2017). Real-time optimized biofeedback utilizing sport techniques (ROBUST): a study protocol for a randomized controlled trial. BMC Musculoskeletal Disord. 18, 71. doi: 10.1186/s12891-017-1436-1 28173788 PMC5297146

[B50] Tong T. K. Wu S. Nie J. (2014). Sport-specific endurance plank test for evaluation of global core muscle function. Phys. Ther. Sport 15, 58–63. doi: 10.1016/j.ptsp.2013.03.003 23850461

[B51] Trovato B. Roggio F. Sortino M. Zanghì M. Petrigna L. Giuffrida R. . (2022). Postural evaluation in young healthy adults through a digital and reproducible method. J. Funct. Morphology Kinesiology 7, 98. doi: 10.3390/jfmk7040098 36412760 PMC9680464

[B52] Wachholz F. Tiribello F. Mohr M. van Andel S. Federolf P. (2020). Adolescent awkwardness: Alterations in temporal control characteristics of posture with maturation and the relation to movement exploration. Brain Sci. 10, 216. doi: 10.3390/brainsci10040216 32260555 PMC7226109

[B53] Wang Z. Chen N. Cao S. Gao L. Geok S. K. Liu J. (2025). The effects of balance training on physical fitness and skill-related performance in basketball players: a systematic review. BMC Sports Sci. Med. Rehabil. 17, 108. doi: 10.1186/s13102-025-01164-9 40312426 PMC12044794

[B54] Williams M. D. Ramirez-Campillo R. Chaabene H. Moran J. (2021). Neuromuscular training and motor control in youth athletes: a meta-analysis. Perceptual Motor Skills 128, 1975–1997. doi: 10.1177/00315125211029006 34293993 PMC8414837

[B55] Yalfani A. Mohamad Khani M. Ahmadi M. Asgarpoor A. (2023). The effect of core stability exercises combined with abdominal hollowing on postural balance in patients with non-specific chronic low back pain: a randomized controlled trial. فیزیک درمانی-نشریه تخصصی فیزیوتراپی 13, 165–174. Physical Therapy - Specialized Physiotherapy Journal, in Persian.

[B56] Zemková E. Zapletalová L. (2022). The role of neuromuscular control of postural and core stability in functional movement and athlete performance. Front. Physiol. 13, 796097. 35283763 10.3389/fphys.2022.796097 PMC8909639

